# Chromatin organization and cytological features of carnivorous *Genlisea* species with large genome size differences

**DOI:** 10.3389/fpls.2015.00613

**Published:** 2015-08-20

**Authors:** Trung D. Tran, Hieu X. Cao, Gabriele Jovtchev, Petr Novák, Giang T. H. Vu, Jiří Macas, Ingo Schubert, Joerg Fuchs

**Affiliations:** ^1^Department of Breeding Research, Leibniz Institute of Plant Genetics and Crop Plant Research (IPK)Gatersleben, Germany; ^2^Institute of Plant Molecular Biology, Biology Centre of the Czech Academy of SciencesČeské Budějovice, Czech Republic; ^3^Central European Institute of Technology and Faculty of Science, Masaryk UniversityBrno, Czech Republic

**Keywords:** *Genlisea*, chromosome number, epigenetic marks, FISH, rDNA, repetitive DNA sequences, single copy probes, karyotyping

## Abstract

The monophyletic carnivorous genus *Genlisea* (Lentibulariaceae) is characterized by a bi-directional genome size evolution resulting in a 25-fold difference in nuclear DNA content. This is one of the largest ranges found within a genus so far and makes *Genlisea* an interesting subject to study mechanisms of genome and karyotype evolution. *Genlisea nigrocaulis*, with 86 Mbp one of the smallest plant genomes, and the 18-fold larger genome of *G. hispidula* (1,550 Mbp) possess identical chromosome numbers (2*n* = 40) but differ considerably in chromatin organization, nuclear and cell size. Interphase nuclei of *G. nigrocaulis* and of related species with small genomes, *G. aurea* (133 Mbp, 2*n* ≈ 104) and *G. pygmaea* (179 Mbp, 2*n* = 80), are hallmarked by intensely DAPI-stained chromocenters, carrying typical heterochromatin-associated methylation marks (5-methylcytosine, H3K9me2), while in *G. hispidula* and surprisingly also in the small genome of *G. margaretae* (184 Mbp, 2*n* = 38) the heterochromatin marks are more evenly distributed. Probes of tandem repetitive sequences together with rDNA allow the unequivocal discrimination of 13 out of 20 chromosome pairs of *G. hispidula*. One of the repetitive sequences labeled half of the chromosome set almost homogenously supporting an allopolyploid status of *G. hispidula* and its close relative *G. subglabra* (1,622 Mbp, 2*n* = 40). In *G. nigrocaulis* 11 chromosome pairs could be individualized using a combination of rDNA and unique genomic probes. The presented data provide a basis for future studies of karyotype evolution within the genus *Genlisea*.

## Introduction

The bladderwort family, Lentibulariaceae, belonging to the eudicot order Lamiales comprises three genera of distinct morphology, *Utricularia* (bladderworts), *Pinguicula* (butterworts), and *Genlisea* (corkscrew plants; Mueller et al., 2003, 2006). The more than 300 different species of the *Lentibulariaceae* are small, herbaceous and predominantly hydrophytes or aquatic (*Utricularia*) plants. All species within this family are carnivorous and each of the three genera developed a distinct trapping mechanism. *Pinguicula* species use sticky, glandular leaves (flypaper traps) to catch small insects. *Utricularia* species have subterraneous leaves forming unique bladder-shaped suction traps to catch mainly aquatic animals and phytoplankton. The genus *Genlisea* developed lobster pot traps of corkscrew-like bundles of root-like subterraneous and chlorophyll-free leaves to attract and entrap a wide spectrum of prokaryotes and small eukaryotes (Cao et al., 2015b). The genus *Genlisea* comprises at least 29 species distributed in South and Central America and in Africa (Fleischmann, 2012). The scientific interest in this genus increased rapidly since Greilhuber et al. (2006) discovered that some of its members possess the smallest nuclear genome size so far recorded for Angiosperms. *Genlisea* *aurea* and *G. margaretae* were described to have a genome size of 63.6 and 63.4 Mbp/1C, respectively. Thus, the genome of *G. aurea* (for *G. margaretae* the ultrasmall genome size could not be confirmed; see Fleischmann, 2012; Veleba et al., 2014; and own data) is less than half of that of *Arabidopsis thaliana* (157 Mbp/1C; Bennett et al., 2003), which was for a long time considered to be the smallest angiosperm genome. *G. hispidula* (1,510 Mbp/1C) and *G. subglabra* (1,471 Mbp/1C) were shown to have up to 24-fold larger genomes (Greilhuber et al., 2006; Fleischmann et al., 2014; Veleba et al., 2014).

Another peculiar feature of *Genlisea* is the exceptionally high DNA substitution rate. In comparison to ∼300 other angiosperm genera representing 200 families, *Genlisea* displayed, together with *Utricularia*, the highest mutation frequency in the chloroplast *matK* gene (Mueller et al., 2003). Similarly, Jobson and Albert (2002) reported a much higher nucleotide substitution rate in the *Genlisea* and *Utricularia* clades, compared to *Pinguicula*, for the non-coding plastid intron regions of trnL-F and rps16, the protein coding chloroplast gene rbcL, the mitochondrial gene coxI, and the nuclear 5.8S rDNA. It was speculated that the high mutation frequency might have facilitated the evolution of the unique subterraneous trapping organs in both genera (Fleischmann, 2012).

Cytological data available for *Genlisea* are restricted to chromosome counts. *G. flexuosa, G. lobata, G. metallica, G. uncinata*, and *G. violacea* belonging to subgenus *Tayloria* have 16 relatively large chromosome pairs while 2*n* = 52 for *G. aurea* and 2*n* = 40 for *G. margaretae* and *G. guianensis* of subgenus *Genlisea* represent approximate counts (Greilhuber et al., 2006; Fleischmann, 2012; Fleischmann et al., 2014). for some *Genlisea* species a precise counting is hampered by large numbers of small chromosomes. In addition, polyploid populations seem to occur within some species as presumed from nuclear DNA contents described for *G. aurea* (Albert et al., 2010) and for *G. repens* (Fleischmann et al., 2014). The assumption of *x* = 8 as the basic number (Fleischmann et al., 2014) is a mere speculation as long as chromosome counting data are not supported by genomic results and/or by fluorescence *in situ* hybridization (FISH).

Recently, whole genome sequence data of four species of the Lentibulariaceae became available, three of them having very small genome sizes, *U. gibba* (88.3 Mbp; Ibarra-Laclette et al., 2013), *G. aurea* (63.6 Mbp; Leushkin et al., 2013) and *G. nigrocaulis* (86 Mbp; Vu et al., in review) and one with a significantly larger genome, *G. hispidula* (1,550 Mbp; Vu et al., in review).

**FIGURE 1:**
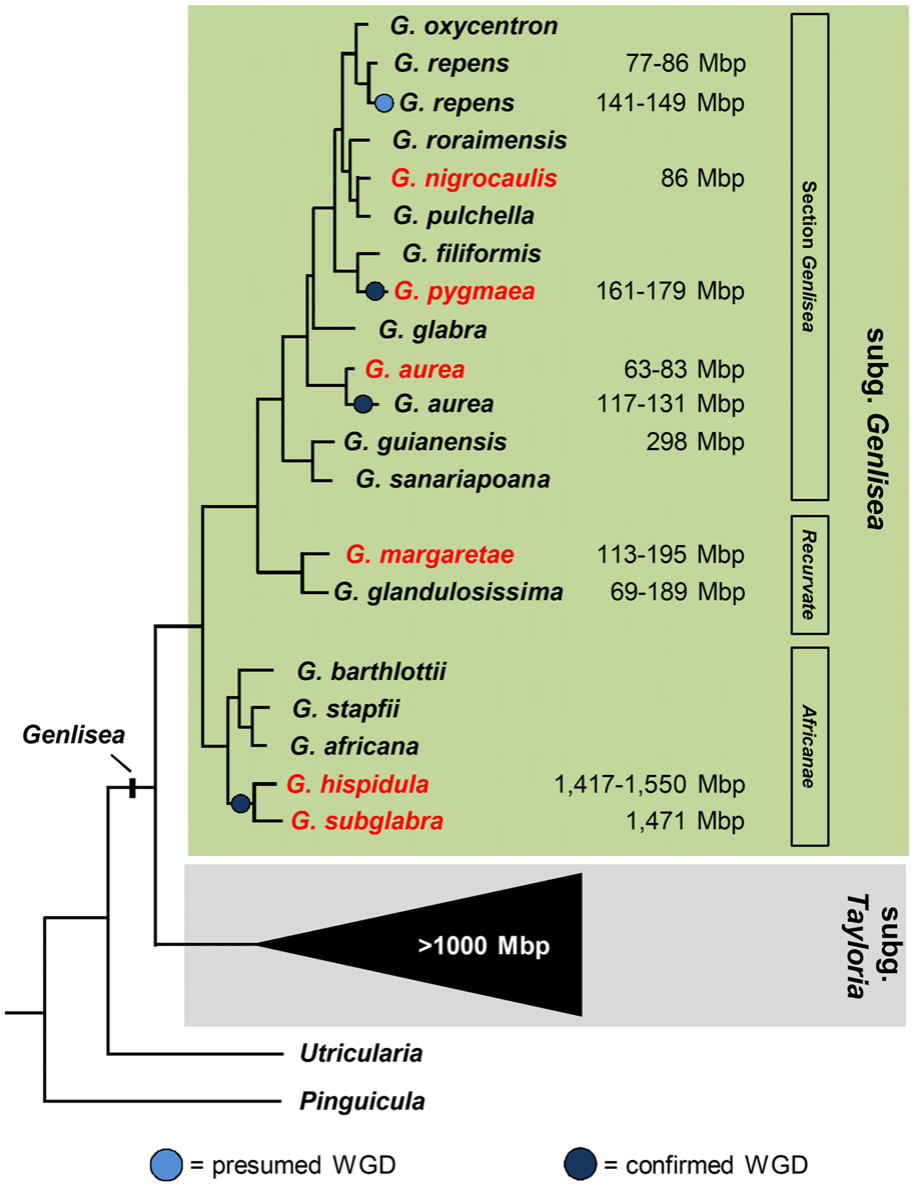
**Phylogenetic relationship of *Genlisea* species belonging to three different sections of subgenus *Genlisea* (modified from Vu et al., in review).** 1C values for *Genlisea* species are from Greilhuber et al. (2006); Fleischmann et al. (2014); Veleba et al. (2014) and Vu et al. (in review). Species used in this study are labeled in red.

Based on available genomic data we present here a cytogenetic characterization of two sections of the subgenus *Genlisea*, represented by *G. nigrocaulis* and *G. hispidula*, which differ significantly in their genome size (**Figure 1**). We determined the chromosome numbers, investigated the appearance of heterochromatin, the sub-nuclear distribution of DNA and histone methylation marks and the chromosomal distribution of rDNA loci in comparison to four other *Genlisea* species possessing either small (*G. aurea*, *G. margaretae*, and *G. pygmaea*) or large (*G. subglabra*) genomes. Furthermore, the chromosomal distribution of retrotransposons and tandem repeats was analyzed by FISH in *G. nigrocaulis* and *G. hispidula.* Based on FISH signals of tandem repeats, 13 chromosome pairs of *G. hispidula* could be individually distinguished. Single copy sequences allowed the discrimination of 11 chromosome pairs of *G. nigrocaulis*.

## Materials and Methods

### Plant Material and Genomic DNA Isolation

Plants of species used in this study (*G. aurea, G. hispidula, G. margaretae, G. nigrocaulis, G. pygmaea*, and *G. subglabra*) were obtained from www.bestcarnivorousplants.com (Ostrava, Czech Republic): *G. aurea*, *G. hispidula*, *G. margaretae*, *G. nigrocaulis*, *G. pygmaea*; www.carnivorsandmore.de (Merzig, Germany): *G. nigrocaulis*, *G. subglabra*, and www.falle.de (Gartenbau Thomas Carow, Nüdlingen, Germany): *G. margaretae*, *G. nigrocaulis*, and cultivated in a green house. Herbarium vouchers of *G. hispidula*, *G. nigrocaulis*, and *G. pygmaea* were deposited at the IPK Gatersleben.

Genomic DNA of *G. nigrocaulis* and *G. hispidula* was isolated using the DNeasy^®^ Plant Mini kit (Qiagen). Concentration and quality of the DNA were estimated using a NanoDrop spectrophotometer (Thermo Scientific) and by 1% agarose-gel electrophoresis.

### Flow Cytometric Genome Size Determination

Genome size measurements were performed according to Fuchs et al. (2008) using either a FACStar^PLUS^ or a FACSAria IIu flow sorter (BD Biosciences). For *G. aurea*, *G. margaretae*, *G. nigrocaulis*, and *G. pygmaea*, *A. thaliana*, ecotype ‘Columbia’ (2C = 0.32 pg; Bennett et al., 2003) and for *G. hispidula* and *G. subglabra*, *Raphanus sativus* ‘Voran’ (IPK gene bank accession number RA 34; 2C = 1.11 pg, Schmidt-Lebuhn et al., 2010), was used as internal reference standard. The absolute DNA contents were calculated based on the values of the G1 peak means.

### Cell and Nuclear Volume Measurements

Upper epidermal layers from healthy young leaves of *G. nigrocaulis* and *G. hispidula* were dissected in a drop of water under a stereomicroscope (Zeiss Stemi 2000, Jena, Germany). After staining of fresh and unfixed tissues with 4,6-diamidino-2-phenylindole (DAPI, 100 ng/ml in water) for 10 min, 3-dimensional images of epidermis cells and their nuclei were acquired using a Zeiss epifluorescence microscope (Axiophot), equipped with a 3CCD Sony color camera (DXC-950P). Thirty cell and nuclear volumes of both species were measured from stack images using the 3D measurement DOMLaycheck software (Schwertner GbR, Jena, Germany). The two-sample *t*-test, assuming equal variances, was used for comparison of values for both species.

### Preparation of Nuclei and Chromosomes

Interphase nuclei from leaves were isolated after formaldehyde fixation, flow-sorted and dropped on slides as described (Lysak et al., 2006b). Slides were stored at -20°C until use.

Chromosome preparations were performed according to Lysak et al. (2006b) with some modifications. For accumulation of dividing cells either young leaves (mitotic cells) or flower buds (mitotic and meiotic cells) were treated with 0.02 M 8-hydroxylquinonline for 2 h at room temperature followed by 4 h at 4°C. After washing in distilled water, the material was fixed in ethanol: glacial acetic acid (3:1) for at least 24 h at room temperature and either used immediately or stored at 4°C for several days. After washing three times in citrate buffer (10 mM sodium citrate, pH 4.5) for 5 min each, the plant tissue was digested in 2% pectinase and 2% cellulase, (w/v) in citrate buffer, at 37°C, for 10 min (young leaves) or 15 min (flower buds). After stopping the digestion by adding ice-cold citrate buffer, the plant tissue was squashed on microscopic slides in a drop of 75% glacial acetic acid. Slides were frozen in liquid nitrogen, rinsed in 2 × SSC, dehydrated in an ethanol series (70, 90, and 96%) and air-dried. Before using them for FISH, the quality of spreading was evaluated by DAPI staining (10 μg/ml in VectaShield). Only slides with more than 10 well-spread metaphases were stored at 4°C and used for FISH.

### Fluorescence *in Situ* Hybridization (FISH) and Immunostaining

To generate FISH probes, *Genlisea*-specific tandem repeats and mobile elements, identified by graph-based clustering analysis, and *G. nigrocaulis*-specific single copy sequences (Vu et al., in review) were PCR amplified with sequence-specific primers (**Table 1**) using GoTag Kit (Promega) or, for fragments longer than 4 kb, Phusion High Fidelity DNA Polymerase (Thermo Scientific). PCR products were analyzed by 1% agarose-gel electrophoresis. Repetitive sequences were further cloned by pGEM-T Easy Vector Systems (Promega) and confirmed by Sanger sequencing. The *A. thaliana* BAC clone T15P10 was used as 45S rDNA probe and 5S rDNA-specific probes were PCR-amplified from genomic DNA of *G. nigrocaulis* using degenerate primers (**Table 1**). Probes were labeled by nick translation (Lysak et al., 2006b) or by PCR (Ali et al., 2005) using either biotin-dUTP, digoxigenine-dUTP (Roche), TexasRed-dUTP, Alexa 488-dUTP (Life Technologies), or Cy3-dUTP (Amersham). FISH including post-hybridization washing under either normal (42°C, for repetitive DNA probes) or higher (50°C, for single copy DNA probes) stringency was performed according to Lysak et al. (2006b). For chromosome individualization, sequential FISH experiments were carried out after probe-stripping from previous hybridization according to Shibata et al. (2009).

**Table 1:** Primer combinations used to amplify unique and repeated DNA elements of *Genlisea nigrocaulis* (Gn) and *G. hispidula* (Gh).

Primer	Primer sequence (5-3)	Product
Gn_v4s196_b1	F: GCAGAGCAAAATCCGGAAAC R: GGCTTCGGCTAATGGACTTG	Single copyfragment of 8.3 kb
Gn_v4s130_z1	F: TACGCTCTGCATTGGGAGTC R: TACGGAAACACCGAACACAA	Single copy fragment of 9.1 kb
Gn_v4s15_p4	F: GGTCATAATTACGGAAGTCGATCC R: GAAACCTGTTTCGGAGAAATCACT	Single copy fragment of 10.4 kb
Gn_v4s56_p44	F: CGTCTGTAGAATTTGAGCAGCGAG R: GCTACTTCATTTGCGGGTGGATAAG	Single copy fragment of 10.5 kb
Gn_v4s2_p6	F: GCCGAAGCGTCATTTACTCACTAC R: CAATCCTCTCCAACGCATCTCTTAC	Single copy fragment of 10.7 kb
Gn_v4c12_p4	F: TGAGTGGTCAAAGAAGACAGGAAG R: ATTTCCGTTAGCGTAGATTCAAGC	Single copy fragment of 8.3 kb
Gn_v4.2s58_4	F: AGTGATGGAAGTGACTCCAGTGAG R: TAATTTCGCTCTCTTGCTGCATAC	Single copy fragment of 9.3 kb
Gn_v4s17_p2	F: ACTCAATCCGGTTCCTGTAAGTTC R: AGTTCATCCTCTGATGGCCTTAAC	Single copy fragment of 10.3 kb
Gn_v4s19_p2	F: CCCAGATGAGAGCAATTTGTATTG R: AACGCATTTCATAGATGAGGATTG	Single copy fragment of 8.5 kb
Gn7c161	F: GCCTTATTATGCATCAAATAGCTTC R: GCAATTGGATCCTTTAATAACCTC	Tandem repeat of 161 bp motif
Gn44c19	F: TTTATTATTTCAGTGTCGGAATGAC R: AATATACGTCATGGAATCAAGATAATG	Tandem repeat of 144 bp motif
Gn10c83	F: GTATATATGTACCGCTTGTGCTCAG R: AACTATATCGTTCAGGCATATGAAAC	*Ty1/copia* element *Bianca* (1,730 bp)
Gh14c16	F: ATAAACACTGATTTCTACCCACCA R: ATGAGTTCTTACACTGATTTCTACCTG	Tandem repeat of 60 bp motif
Gh250c46	F: GAGCTCGTTCCTGATCAGTCC R: ACTGGAAGAATCTTTCCGATCTC	Tandem repeat of 74 bp motif
Gh45c31	F: TCGAAGAGATCGGATAGATAGAATC R: GTTTGTTCAGTTCAACATTTGAGG	Tandem repeat of110 bp motif
Gh80c174	F: TTGAGCTCGATCAGTTTCACC R: GAGATCAAATAGATTGAATCATCCAG	Tandem repeat of 112 bp motif
Gh336c35	F: ACCACGTGGCCGATCTGT R: AGATCGGTCTAGGCGTGGAA	Tandem repeat of 146 bp motif
Gh19c56	F: GTTTTGCGGTAAGTAATCCAATG R: TGCAATAATTCGACTACGAAATCAC	Tandem repeat of 900 bp motif
5SrDNA	F: GTGCGATCATACCAGCRKTAATRCACCGG R: GAGGTGCAACACGAGGACTTCCCAGGRGG	*Genlisea* specific 5S rDNA

For immunostaining of histones, flow-sorted nuclei preparations were baked at 60°C for 15 min. Then slides were incubated in blocking buffer (5% horse serum, 3% BSA in 1x PBS) at 37°C for 1 h, shortly washed in PBS and incubated with the primary antibody at 4°C for 16 h in a humid chamber. Rabbit antibodies against H3K4me2 and H3K9me2 (Millipore, cat-No. 07-030 and 07-441, dilution 1:200) were used. Subsequently, slides were washed in PBS three times for 10 min each and then incubated with Cy3-conjugated anti rabbit antibody (dilution 1:200) in a humid chamber at 37°C for 1 h. After final washes in PBS (three times 10 min each), the slides were counter-stained with DAPI. For detection of DNA methylation, nuclei were post-fixed and denatured as described for FISH and afterward incubated with a mouse antibody against 5-methylcytosine (Eurogentec, cat-No. MMS-900P-A), followed by an Alexa 488-conjugated anti mouse (1:100) antibody.

### Microscopy and Image Processing

Fluorescence *in situ* hybridization and immunostaining preparations were analyzed under a Zeiss Axioplan 2 epifluorescence microscope equipped with a cooled CCD camera (Diagnostic Instruments, Inc.) using a 100× objective. Fluorescence images for each fluorochrome were captured separately using appropriate filter combinations. The images were pseudo-colored, merged and processed (brightness and contrast adjustment only) with Adobe Photoshop software (Adobe Systems).

## Results

### *G. nigrocaulis* and *G. hispidula* Differ in Genome, Nucleus and Cell Size

**FIGURE 2:**
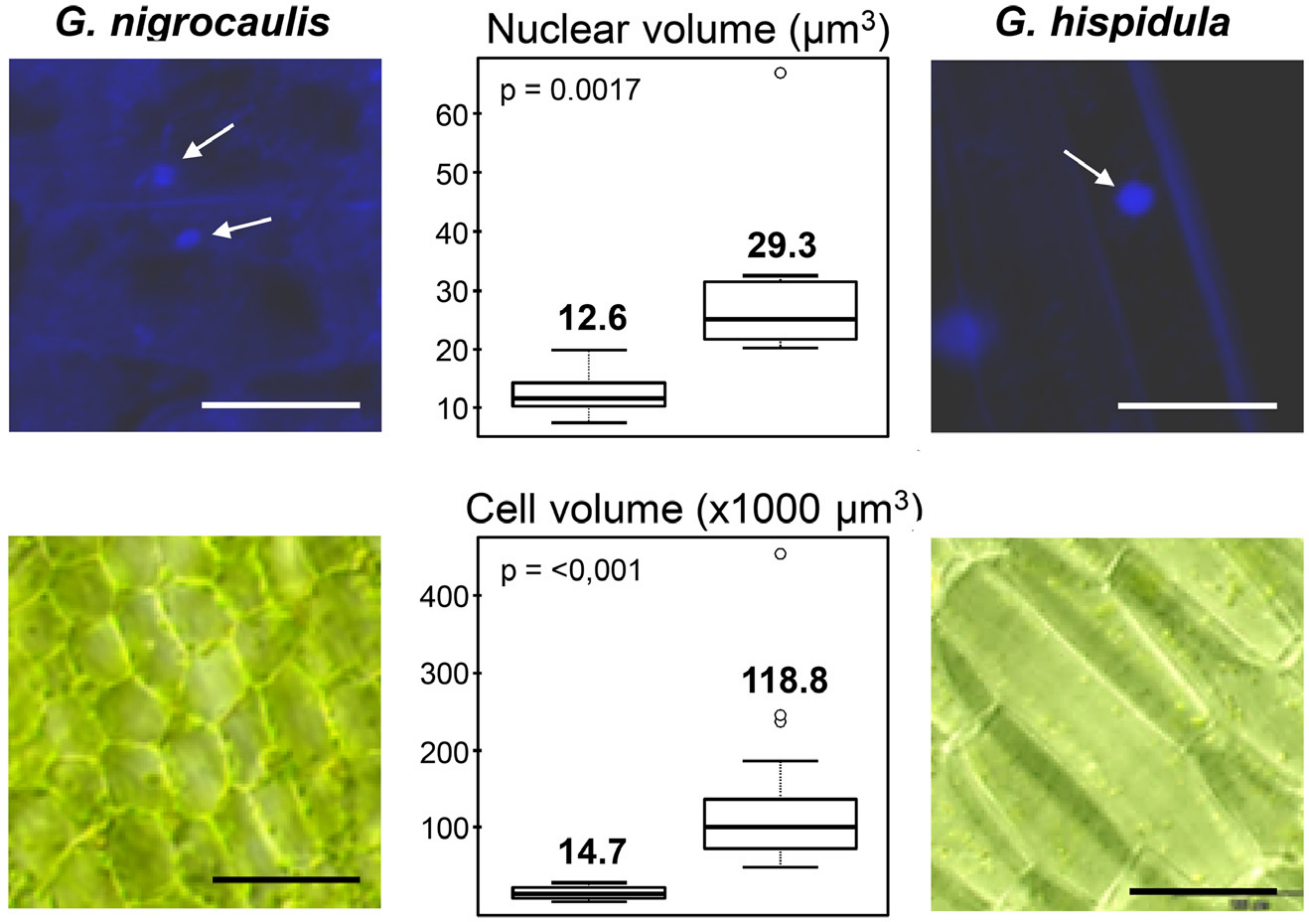
**Significant (*P* < 0.01%) cell and nuclear size differences occur between *Genlisea nigrocaulis* and *G. hispidula* (arrows indicate nuclei).** Bars: 100 μm in nuclear images (above) and 20 μm in cell images (below).

**FIGURE 3:**
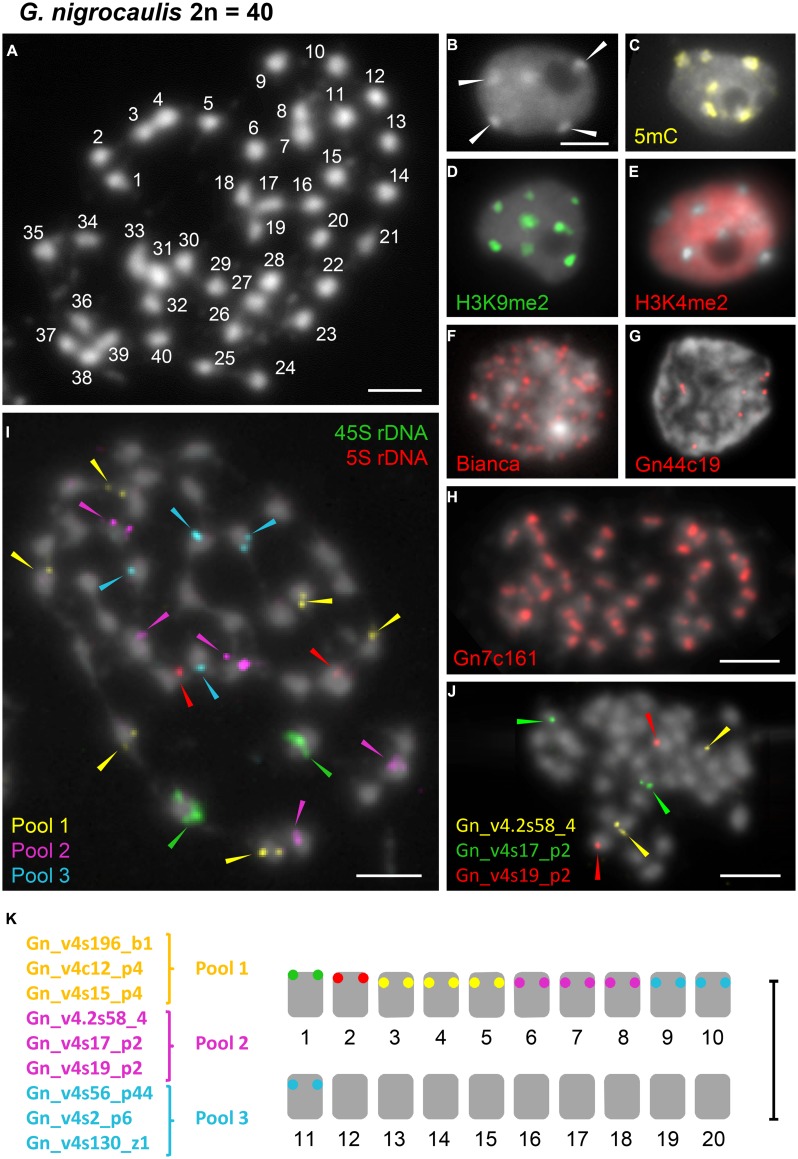
**Distribution of immunosignals as well as of the retroelement *Bianca*, of tandem repeats and single copy sequences within nuclei or on chromosomes of *G. nigrocaulis.* (A)** Chromosome complement of *G. nigrocaulis* with 2*n* = 40 chromosomes. **(B–E)** Interphase nuclei after DAPI-staining **(B)** and immunostaining with antibodies against 5-mC **(C)**, H3K9me2 **(D)**, and H3K4me2 **(E)** to indicate the distribution of eu- and heterochromatin. Arrows in **(B)** denote heterochromatic chromocenters. **(F,G)** Interphase nuclei after FISH with the highly abundant *Ty1/copia* element *Bianca* **(F)** and one of the two abundant tandem repeats Gn44c19 (144 bp, **G**). **(H)** FISH with the most abundant tandem repeat Gn7c161 on metaphase nuclei resulted in a single hybridization signal per chromosome indicating that Gn7c161 might be a centromere-associated sequence. **(I)** FISH-based karyotype in conjunction with rDNA probes using nine single-copy probes which were divided into three pools. Each pool comprised three probes which were shown to label one different chromosome pair each in separate FISH experiments. Additional images confirming the separate localization of all three probes of pool 3 (cyan) are provided in Supplementary Figure S4. **(J)** Example of individual probe testing before combination into three pools. Probes from pool 2 are shown. **(K)** Karyogram of *G. nigrocaulis* with 11 distinguishable chromosome pairs. Bars = 3 μm.

**FIGURE 4:**
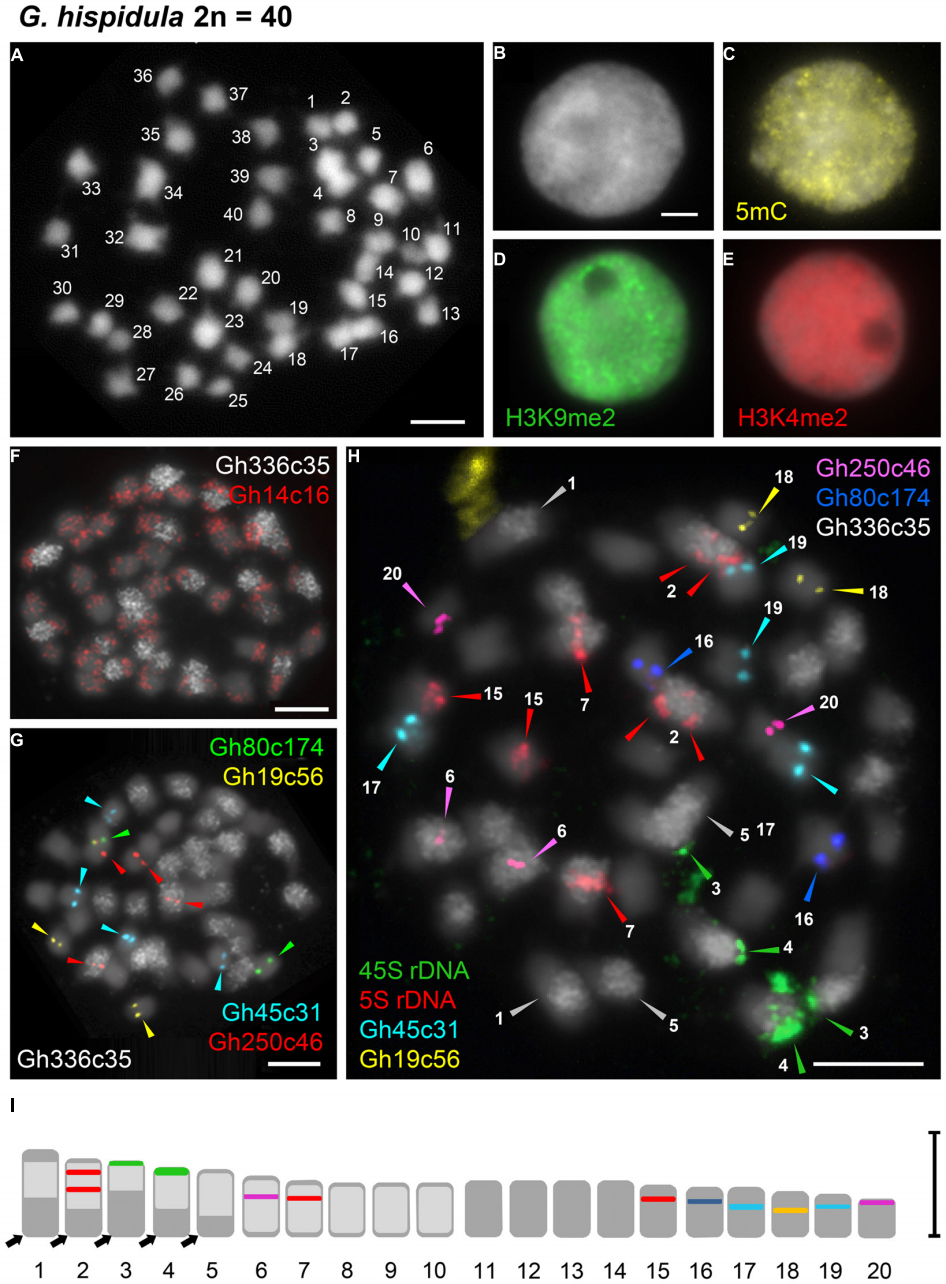
**Distribution of immunosignals and tandem repeats on nuclei or chromosomes of *G. hispidula*. (A)** Chromosome complement of *G. hispidula* with 2*n* = 40 chromosomes. **(B–E)** Interphase nuclei after DAPI-staining **(B)** and immunostaining with antibodies against 5-mC **(C)**, H3K9me2 **(D)**, and H3K4me2 **(E)** to indicate the distribution of eu- and heterochromatin. **(F)** Two tandem repeats yielding dispersed FISH signals; Gh14c16 labels each chromosome pair (red) and Gh336c35 strongly accumulates on 10 of the 20 chromosome pairs (white). **(G)** Tandem repeats with distinct FISH signals on one (Gh19c56, yellow; Gh80c174, green) or two (Gh250c46, red; Gh45c31, cyan) chromosome pairs. With the exception of one locus of Gh250c46 all loci were found in the chromosome set with low abundance of Gh336c35 (white). **(H)** Metaphase after sequential FISH with seven tandem repetitive sequences including the rDNA probes allowing the unequivocal discrimination of 13 chromosome pairs (arrow heads). **(I)** Karyogram of *G. hispidula* based on seven metaphases indicating the 13 distinguishable chromosome pairs (1–7, 15–20); chromosome numbers and colors of markers correspond to those denoted in **(H)**. The arrows indicate regions free of Gh336c35 that are assumed to result from reciprocal exchanges between homeologous chromosomes of the parental genomes and subsequent biased segregation of translocation products. Bars = 3 μm.

The nuclear DNA content of *G. nigrocaulis* was estimated to be 0.088 pg/1C corresponding to 86 Mbp according to the conversion proposed by Dolezel et al. (2003), while that of *G. hispidula* was estimated to be 1,590 pg/1C corresponding to 1,550 Mbp (Vu et al., in review). This 18-fold genome size difference between both species is also reflected by a significantly larger average volume of nuclei (29.3 μm^3^ vs. 12.6 μm^3^ = 2.3x) and of cells (118,800 μm^3^ vs. 14,700 μm^3^ = 8.08x) in *G. hispidula* (**Figure 2**). Nevertheless, both species have 2*n* = 40 chromosomes with an average length of 0.5–1 μm in *G. nigrocaulis* and ∼2.5 μm in *G. hispidula* (**Figures 3A and 4A**; Vu et al., in review). Furthermore, we estimated the genome sizes and counted the chromosome numbers of *G. aurea* (133 Mbp/2*n* ≈ 104), *G. margaretae* (184 Mbp/2*n* = 38), *G. pygmaea* (179 Mbp/2*n* = 80), and *G. subglabra* (1,622 Mbp/2*n* = 40; Supplementary Figure S1).

### The Small Genome of *G. nigrocaulis* Displays Distinct Heterochromatin Features

DAPI-staining of flow-sorted interphase nuclei revealed surprisingly distinct heterochromatic chromocenters in the small genome of *G. nigrocaulis* (**Figure 3B**), while nuclei of the 18-fold larger genome of *G. hispidula* displayed a nearly homogeneous DAPI staining without conspicuous heterochromatin clusters (**Figure 4B**). Immunolabelling of interphase nuclei using antibodies against 5-methylcytosine (5 mC) was performed to investigate the subnuclear distribution of DNA methylation. While nuclei of *G. hispidula* revealed a dispersed DNA methylation throughout the entire nucleus (**Figure 4C**), nuclei of *G. nigrocaulis* showed an accumulation of signals at the heterochromatic chromocenters (**Figure 3C**). A similar signal pattern was obtained using antibodies against H3K9me2, a modification that was previously identified as a conserved heterochromatin-associated mark in plants (Fuchs and Schubert, 2012; **Figure 3D** and Supplementary Figure S2D). The typical euchromatin-associated mark H3K4me2 labeled in *G. nigrocaulis* exclusively the euchromatic regions (**Figure 3E**). In *G. hispidula* these marks were found in most nuclei homogeneously distributed over the entire chromatin (**Figures 4D,E**).

Other species with small genomes, *G. aurea* and *G. pygmaea*, revealed, comparable to *G. nigrocaulis*, heterochromatic chromocenters and the same nuclear distribution of histone and DNA modification marks in interphase nuclei, while the genome of *G. margaretae* (184 Mbp) surprisingly resembled that of *G. hispidula* (Supplementary Figure S2).

### Chromosomal Localization of Diverse Tandem Repeats Allows Distinguishing of 13 *G. hispidula* Chromosome Pairs

The 45S and the 5S rDNA have been localized on one chromosome pair each in *G. nigrocaulis* (**Figure 3I** and Supplementary Figure S3A), while they were found on two and three chromosome pairs, respectively, in *G. hispidula* (**Figure 4H** and Supplementary Figure S3E). One of the *G. hispidula* chromosome pairs harbors two 5S rDNA loci (**Figure 4H** and Supplementary Figure S3E).

Graph-based clustering analysis according to Novak et al. (2010) revealed the constitution of other repetitive DNA sequences in both genomes (Vu et al., in review). FISH probes derived from consensus sequences of the different repeat families (**Table 1**) were used to investigate their subnuclear and/or chromosomal distribution.

In *G. hispidula*, the six most abundant tandem repeats (besides the rDNA), Gh14c16, Gh250c46, Gh45c31, Gh80c174, Gh336c35, and Gh19c56, have monomer lengths of 60, 74, 110, 112, 146, and 900 bp, respectively. Two of them revealed dispersed FISH signals: Gh14c16 revealed signals on each chromosome pair with a tendency to accumulate toward the chromosome ends (**Figure 4F**), while Gh336c35 showed a high accumulation on 10 of 20 chromosome pairs (**Figure 4F**). Three tandem repeats were found exclusively in the chromosome set with low abundance of Gh336c35: Gh19c56 and Gh80c174 revealed distinct signals on one chromosome pair each (**Figure 4G**); Gh45c31 showed signals on two chromosome pairs, one signal pair slightly weaker than the other (**Figure 4G**). Gh250c46 revealed signals on two chromosome pairs, one with high and one with low Gh336c35 abundance (**Figure 4G**). Sequential multicolor-FISH using all tandem repeats that label one or two distinct chromosome pairs, together with Gh336c35 and the rDNA, on one metaphase plate allowed to discriminate 13 of the 20 chromosome pairs of *G. hispidula* (**Figures 4H,I**).

PCR amplification confirmed the presence of the six *G. hispidula* tandem repeats in the closely related genome of *G. subglabra*. FISH using Gh336c35 as probe labeled, similar as in *G. hispidula*, half of the chromosome set (Supplementary Figure S5). Whereas the Gh45c31 tandem repeat showed at maximum two distinct signals instead of four on all evaluated interphase nuclei (Supplementary Figure S5). The 45S and the 5S rDNA have been localized on two and four chromosomes, respectively. Similar as in *G. hispidula*, one chromosome pair harbors two 5S rDNA loci in *G. subglabra* (2*n* = 40; Supplementary Figure S3F).

### rDNA and Single Copy Sequences Discriminate 11 Chromosome Pairs of *G. nigrocaulis* and Homeologs of the Tetraploid *G. pygmaea* which Shares Even Repetitive Sequences with *G. nigrocaulis*

In *G. nigrocaulis*, one of the few abundant and probably still active retroelements is the *Ty1*/*copia* element *Bianca* (Vu et al., in review). FISH with this element yielded many signals throughout the nucleus (**Figure 3F**). In the genome of *G. nigrocaulis* only two major families of tandem repeats could be identified. Gp44c19 with a monomer length of 144 bp revealed up to 10 signals of varying intensity per interphase nucleus (**Figure 3G**). By far the most abundant tandem repeat in *G. nigrocaulis* is a 161 bp repeat (Gp7c161). FISH yielded strong hybridization signals on each metaphase chromosome (**Figure 3H**) suggesting this sequence is a (peri)centromere-associated repeat, as was confirmed by co-localization with a centromere-specific antibody (Tran et al., in review). Based on FISH signals for 45S and 5S rDNA, which label one chromosome pair each (Supplementary Figure S3A), and nine single copy probes (Vu et al., in review) 11 chromosome pairs of *G. nigrocaulis* could be identified (**Figures 3I–K** and Supplementary Figure S4).

Similar as in *G. nigrocaulis*, the centromeric 161 bp tandem repeat Gn7c161 co-localized with heterochromatic chromocenters in interphase nuclei of *G. pygmaea* (Tran et al., in review). The most abundant and dispersed *Ty1/copia* retroelement *Bianca* of *G. nigrocaulis*, which is not detectable within the *G. hispidula* genome, yields FISH signals on *G. pygmaea* nuclei (Supplementary Figure S6A). Possibly *Bianca* entered the common ancestor of both related species *via* horizontal transfer which seems to occur more frequently than previously assumed (El Baidouri et al., 2014). Furthermore, FISH with total genomic DNA of *G. nigrocaulis* and an excess of unlabeled 161 bp repeat sequence on nuclei of *G. pygmaea* revealed dispersed signals of similar intensity as in *G. nigrocaulis* throughout the entire genome except at chromocenters, emphasizing the close relationship of both species (Supplementary Figures S6B,C).

The 45S and 5S rDNA sequences have been localized on one chromosome pair each in *G. nigrocaulis* and *G. pygmaea*, although FISH with unique genomic probes indicated a polyploidization event in *G. pygmaea* (possibly autotetraploidy) after the split of the two species (Vu et al., in review). Also in *G. margaretae* we found one chromosome pair each carrying the 45S and 5S rDNA (Supplementary Figures S3B,D), while two chromosome pairs harbor FISH signals for 45S as well as for 5S rDNA in the tetraploid population of *G. aurea* (Albert et al., 2010; Supplementary Figure S3C).

## Discussion

Despite an 18-fold genome size difference, *G. nigrocaulis* and *G. hispidula* share the same chromosome number (2*n* = 40). The predicted gene copy number and a high frequency of single-nucleotide polymorphisms with a ∼1:1 allele ratio indicated that *G. hispidula* is allotetraploid (Vu et al., in review). Allotetraploidy is further supported by the FISH pattern of the repetitive sequence probe Gh336c35 which resulted in signals on 10 of the 20 chromosome pairs. Based on the chromosome number of 2*n* = 40, a dysploid chromosome number reduction has to be assumed either for both ancestor species, or after whole genome duplication in *G. hispidula*, similarly as shown for Australian Brassicaceae species (Mandakova et al., 2010). However, the FISH signals for Gh336c35 suggest that dysploid chromosome number reduction might have occurred already within the ancestral species of the *G. hispidula* lineage. Such chromosome number reduction is often the result of reciprocal translocations with terminal breakpoints which combine two linkage groups into one large chromosome, while the second translocation product is very small and prone to get lost during meiosis (Schubert and Lysak, 2011). The absence of Gh336c35 signals in some terminal regions of chromosome 1–5 (**Figures 4H,I**), might be due to reciprocal exchanges between homeologs (among chromosomes 11–20) and subsequent segregation bias (Wicker et al., in review) within allotetraploid *G. hispidula*. Alternatively, the exchanged segments were of unequal size and very small Gh336c35-rich regions, transferred to chromosomes of the originally Gh336c35 signal-free complement, are not detectable by FISH. A BAC tiling path might enable to identify chromosome homeology between *Genlisea* species and to trace the route of karyotype evolution within this genus *via* interspecific chromosome painting as was shown for Brassicaceae (Lysak et al., 2006a).

The difference in genome size between both species is also reflected by a 2.3× larger nuclear and an 8× larger cellular volume in *G. hispidula* versus *G. nigrocaulis*. This is in line with previous investigations showing for vacuole-free epidermal cells of endopolyploid and non-endopolyploid plant species a positive correlation between DNA content, nuclear and cellular volume (Jovtchev et al., 2006).

A subnuclear clustering of the heterochromatin-specific chromatin mark H3K9me2, as previously observed for the small genome of *A. thaliana* (Soppe et al., 2002), was also found for *G. nigrocaulis* and in other small genome species such as *G. aurea* and *G. pygmaea*. Although also possessing a small genome, similar to that of *A. thaliana*, *G. margaretae* showed an exceptionally homogenous distribution of this mark, resembling that typical for large genomes (Supplementary Figure S2). In larger genomes (>500 Mb), heterochromatic marks are often distributed more uniformly because of a higher density of mobile elements to be silenced. Additionally, in some medium-sized genomes and even in small genomes of neotenic plants a lack of pronounced heterochromatic chromocenters may occur (Houben et al., 2003; Cao et al., 2015a). Regarding the absence of pronounced chromocenters and the rather uniform distribution of heterochromatic chromatin marks, the nuclear phenotypes of *G. hispidula* and even *G. margaretae* resemble the observations made for medium-sized genomes (Supplementary Figure S2).

Possessing numerous small chromosomes that lack conspicuous primary constrictions and a clear bi-armed appearance, karyotyping in *Genlisea* species relying on chromosome measurement and chromosome banding is impossible, but FISH can overcome this limitation.

Repetitive DNA, such as tandem repeats and transposable elements, constitutes a considerable part of plant genomes (Lopez-Flores and Garrido-Ramos, 2012). Tandem repeats often facilitate FISH-based chromosome identification. For instance, ribosomal DNAs are the most frequently used probes in initial karyotyping due to their conserved sequence and variable loci number and position (Garcia et al., 2014). The rDNA loci allowed also in *Genlisea* species to distinguish several chromosome pairs and revealed a similar distribution in species possessing similar genome sizes. Furthermore it enabled to differentiate the similar karyotypes of *G. hispidula* and *G. subglabra*. Remarkably, the tetraploid *G. pygmaea* has retained only one set of 45S and 5S rDNA repeats. Other tandem repeat sequences which are species-specific or shared between related species can also be useful for karyotyping. Although more than half of the *G. hispidula* genome was characterized as repetitive DNA (Vu et al., in review), only few tandem repeat sequences were identified. Five of these repeats show chromosome-specific FISH signals which in combination with rDNA probes unequivocally distinguished 13 chromosome pairs of *G. hispidula*. The presence of these tandem repeat sequences in *G. subglabra* together with the shared centromere and telomere sequences (Tran et al., in review) indicate the close relatedness of these two species. The Gh336c35 repeat labeled 10 of the 20 chromosome pairs of *G. hispidula* as well as of *G. subglabra*. Such a signal pattern resembles the results frequently obtained by genomic *in situ* hybridization (GISH) for allopolyloid species with genomic DNA of a species related to one ancestor as probe, and supports the assumption of allotetraploidy for *G. hispidula* (Vu et al., in review) and for *G. subglabra* and indicates a shared ancestor species.

Single-copy sequences together with rDNA probes proved to be helpful for the challenging task to individualize more than half of the 20 small chromosome pairs of *G. nigrocaulis* and to address homeologous chromosomes of *G. pygmaea*. The presented data provide sets of chromosome-specific markers which may serve as an anchor for further cytological analysis within the genus *Genlisea* with the aim to elucidate karyotype evolution during speciation. In conjunction with data on centromere and telomere characterization (Tran et al., in review), these results provide the hitherto most detailed karyotype analyses for *Genlisea* species.

## Author Contributions

IS and JF conceived and coordinated the study. JM participated in study design. TT, HC, and GJ performed the experiments. HC, PN, GV, and JM performed comparative genome analysis. TT, IS, and JF wrote the manuscript with input from HC, PN, and JM. All authors read and approved the final manuscript.

## Conflict of Interest Statement

The authors declare that the research was conducted in the absence of any commercial or financial relationships that could be construed as a potential conflict of interest.

## References

[B1] Albert V. A. Jobson R. W. Michael T. P. Taylor D. J. (2010). The carnivorous bladderwort (*Utricularia*, Lentibulariaceae): a system inflates. *J. Exp. Bot.* 61 5–9. 10.1093/jxb/erp349 20007200

[B2] Ali H. B. Lysak M. A. Schubert I. (2005). Chromosomal localization of rDNA in the Brassicaceae. *Genome* 48 341–346. 10.1139/g04-116 15838557

[B3] Bennett M. D. Leitch I. J. Price H. J. Johnston J. S. (2003). Comparisons with *Caenorhabditis* (approximately 100 Mb) and *Drosophila* (approximately 175 Mb) using flow cytometry show genome size in *Arabidopsis* to be approximately 157 Mb and thus approximately 25% larger than the *Arabidopsis* genome initiative estimate of approximately 125 Mb. *Ann. Bot.* 91 547–557. 10.1093/aob/mcg057 12646499 PMC4242247

[B4] Cao H. X. Vu G. T. H. Wang W. Messing J. Schubert I. (2015a). Chromatin organisation in duckweed interphase nuclei in relation to the nuclear DNA content. *Plant Biol.* 17(Suppl. 1), 120–124. 10.1111/plb.12194 24853858

[B5] Cao H. X. Schmutzer T. Scholz U. Pecinka A. Schubert I. Vu G. T. H. (2015b). Metatranscriptome analysis reveals host-microbiome interactions in traps of carnivorous *Genlisea* species. *Front. Microbiol.* 6:526 10.3389/fmicb.2015.00526 PMC4500957 26236284

[B6] Dolezel J. Bartos J. Voglmayr H. Greilhuber J. (2003). Nuclear DNA content and genome size of trout and human. *Cytometry A* 51 127–128. 10.1002/cyto.a.10013 12541287

[B7] El Baidouri M. Carpentier M. C. Cooke R. Gao D. Lasserre E. Llauro C. (2014). Widespread and frequent horizontal transfers of transposable elements in plants. *Genome Res.* 24 831–838. 10.1101/gr.164400.113 24518071 PMC4009612

[B8] Fleischmann A. (2012). *Monograph of the Genus Genlisea.* Dorset: Redfern Natural History Productions.

[B9] Fleischmann A. Michael T. P. Rivadavia F. Sousa A. Wang W. Temsch E. M. (2014). Evolution of genome size and chromosome number in the carnivorous plant genus *Genlisea* (Lentibulariaceae), with a new estimate of the minimum genome size in angiosperms. *Ann. Bot.* 114 1651–1663. 10.1093/aob/mcu189 25274549 PMC4649684

[B10] Fuchs J. Jovtchev G. Schubert I. (2008). The chromosomal distribution of histone methylation marks in gymnosperms differs from that of angiosperms. *Chromosome Res.* 16 891–898. 10.1007/s10577-008-1252-4 18679813

[B11] Fuchs J. Schubert I. (2012). “Chromosomal distribution and functional interpretation of epigenetic histone marks in plants,” in *Plant Cytogenetics*, eds Bass H. W. Birchler J. A. (New York, NY: Springer), 231–253. 10.1007/978-0-387-70869-0_9

[B12] Garcia S. Galvez F. Gras A. Kovarik A. Garnatje T. (2014). Plant rDNA database: update and new features. *Database (Oxford)* 2014:bau063 10.1093/database/bau063 PMC4075780 24980131

[B13] Greilhuber J. Borsch T. Worberg A. Porembski S. Barthlott W. (2006). Smallest angiosperm genomes found in Lentibulariaceae, with chromosome of bacterial size. *Plant Biol.* 8 770–777. 10.1055/s-2006-924101 17203433

[B14] Houben A. Demidov D. Gernand D. Meister A. Leach C. R. Schubert I. (2003). Methylation of histone H3 in euchromatin of plant chromosomes depends on basic nuclear DNA content. *Plant J.* 33 967–973. 10.1046/j.1365-313X.2003.01681.x 12631322

[B15] Ibarra-Laclette E. Lyons E. Hernandez-Guzman G. Perez-Torres C. A. Carretero-Paulet L. Chang T. H. (2013). Architecture and evolution of a minute plant genome. *Nature* 498 94–98. 10.1038/nature12132 23665961 PMC4972453

[B16] Jobson R. W. Albert V. A. (2002). Molecular rates parallel diversification contrasts between carnivorous plant sister lineages1. *Cladistics* 18 127–136. 10.1111/j.1096-0031.2002.tb00145.x 34911224

[B17] Jovtchev G. Schubert V. Meister A. Barow M. Schubert I. (2006). Nuclear DNA content and nuclear and cell volume are positively correlated in angiosperms. *Cytogenet. Genome. Res.* 114 77–82. 10.1159/000091932 16717454

[B18] Leushkin E. Sutormin R. Nabieva E. Penin A. Kondrashov A. Logacheva M. (2013). The miniature genome of a carnivorous plant *Genlisea aurea* contains a low number of genes and short non-coding sequences. *BMC Genomics* 14:476 10.1186/1471-2164-14-476 PMC3728226 23855885

[B19] Lopez-Flores I. Garrido-Ramos M. A. (2012). The repetitive DNA content of eukaryotic genomes. *Genome Dyn.* 7 1–28. 10.1159/000337118. 22759811

[B20] Lysak M. A. Berr A. Pecinka A. Schmidt R. McBreen K. Schubert I. (2006a). Mechanisms of chromosome number reduction in *Arabidopsis thaliana* and related Brassicaceae species. *Proc. Natl. Acad. Sci. U.S.A.* 103 5224–5229. 10.1073/pnas.0510791103 16549785 PMC1458822

[B21] Lysak M. A. Fransz P. Schubert I. (2006b). Cytogenetic analyses of *Arabidopsis*. *Methods Mol. Biol.* 323 173–186. 10.1385/1-59745-003-0 16739577

[B22] Mandakova T. Joly S. Krzywinski M. Mummenhoff K. Lysak M. A. (2010). Fast diploidization in close mesopolyploid relatives of *Arabidopsis*. *Plant Cell* 22 2277–2290. 10.1105/tpc.110.074526 20639445 PMC2929090

[B23] Mueller K. F. Borsch T. Legendre L. Porembski S. Barthlott W. (2006). Recent progress in understanding the evolution of carnivorous Lentibulariaceae (Lamiales). *Plant Biol.* 8 748–757. 10.1055/s-2006-924706 17203430

[B24] Mueller K. Borsch T. Legendre L. Porembski S. Theisen I. Barthlott W. (2003). Evolution of carnivory in Lentibulariaceae and the Lamiales. *Plant Biol.* 6 477–490. 10.1055/s-2004-817909 15248131

[B25] Novak P. Neumann P. Macas J. (2010). Graph-based clustering and characterization of repetitive sequences in next-generation sequencing data. *BMC Bioinformatics* 11:378 10.1186/1471-2105-11-378 PMC2912890 20633259

[B26] Schmidt-Lebuhn A. N. Fuchs J. Hertel D. Hirsch H. Toivonen J. Kessler M. (2010). An Andean radiation: polyploidy in the tree genus *Polylepis* (Rosaceae, Sanguisorbeae). *Plant Biol.* 12 917–926. 10.1111/j.1438-8677.2009.00297.x 21040307

[B27] Schubert I. Lysak M. A. (2011). Interpretation of karyotype evolution should consider chromosome structural constraints. *Trends Genet.* 27 207–216. 10.1016/j.tig.2011.03.004 21592609

[B28] Shibata F. Sahara K. Naito Y. Yasukochi Y. (2009). Reprobing multicolor FISH preparations in lepidopteran chromosome. *Zoolog. Sci.* 26 187–190. 10.2108/zsj.26.187 19341338

[B29] Soppe W. J. Jasencakova Z. Houben A. Kakutani T. Meister A. Huang M. S. (2002). DNA methylation controls histone H3 lysine 9 methylation and heterochromatin assembly in *Arabidopsis*. *EMBO J.* 21 6549–6559. 10.1093/emboj/cdf657 12456661 PMC136960

[B30] Veleba A. Bures P. Adamec L. Smarda P. Lipnerova I. Horova L. (2014). Genome size and genomic GC content evolution in the miniature genome-sized family Lentibulariaceae. *New Phytol.* 203 22–28. 10.1111/nph.12790 24661198

