# Supplementary material for: Chromatin organization and cytological features of carnivorous Genlisea species with large genome size differences
Source: Front Plant Sci. 2015 Aug 20;6:613. doi: 10.3389/fpls.2015.00613 (PMC4542322; doi:10.3389/fpls.2015.00613)
Supplement: Supplementary file 1 [file Image_1.PDF]

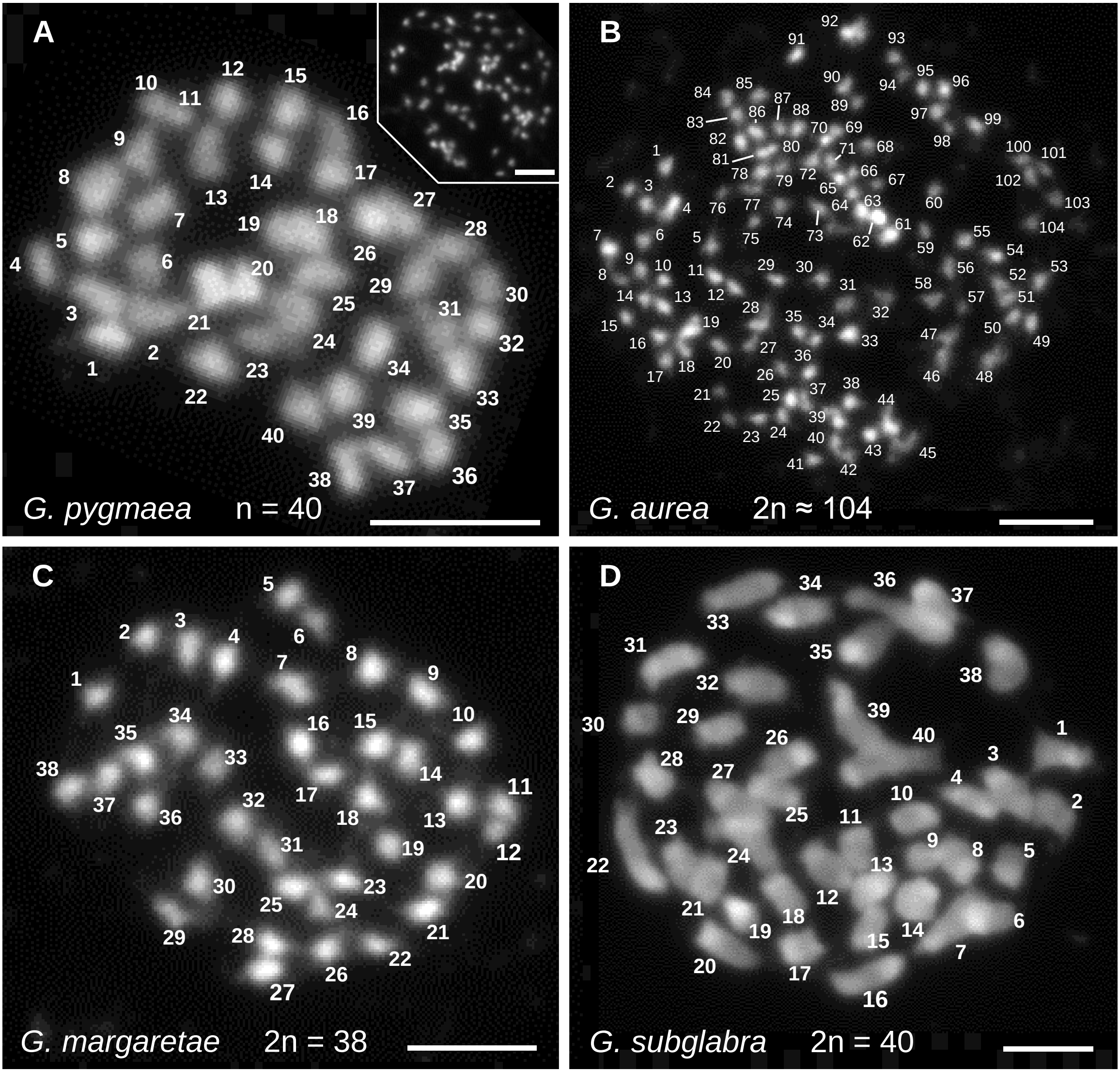

Supplementary figure 1: Chromosome number of some *Genlisea* species representing the three sections of subgenus *Genlisea*. (**A**) 40 meiotic metaphase II chromosomes of *G. pygmaea*. The inset shows a mitotic metaphase. (**B**) Mitotic spreading showing 2n ≈ 104 chromosomes of *G. aurea*. (**C**) 2n = 38 of *G. margaretae*. (**D**) 2n = 40 in *G. subglabra*. Bars = 5 µm.

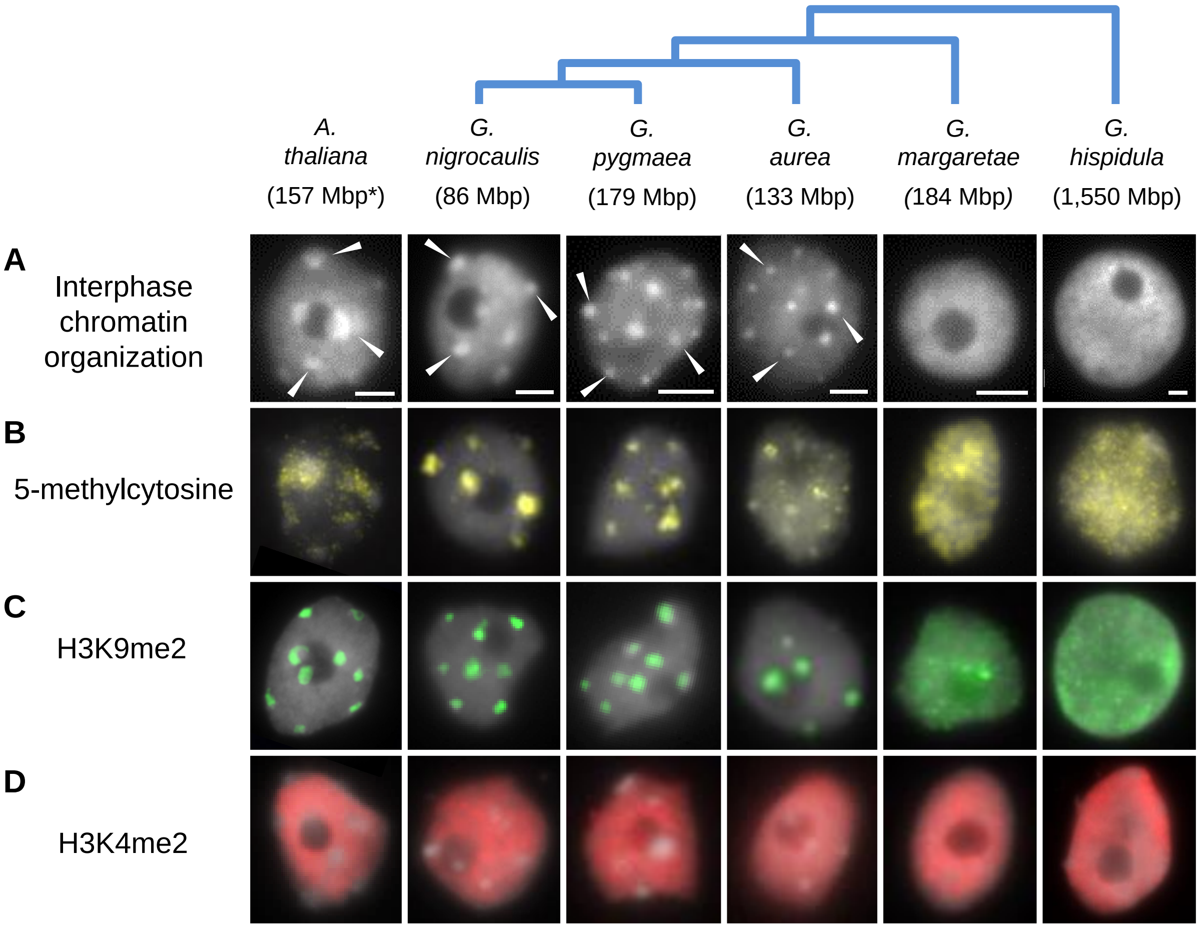

Supplementary figure 2: Heterochromatin organization and distribution of epigenetic marks in *Genlisea* species in comparison to *A. thaliana*. (**A**) Interphase chromatin organization of *Genlisea* species in conjunction with genome size and phylogenetic relationship modified from Vu et al., submitted. Pronounced heterochromatic chromocenters (white arrow heads) can be observed in small genome species as in *A. thaliana* (* Bennett et al., 2003). Bars = 2.5 µm. (**B** and **C**) Distribution of heterochromatin-specific marks (DNA methylation and H3K9me2 in (**B**) and (**C**), respectively) in small genomes resembles to that of *A. thaliana*. (**D**) Dispersed distribution of euchromatin-specific histone mark H3K4me2 except at heterochromatic chromocenters in small genomes. Note that *G. hispidula* and *G. margaretae* show dispersed signal patterns resembling that of larger genomes.

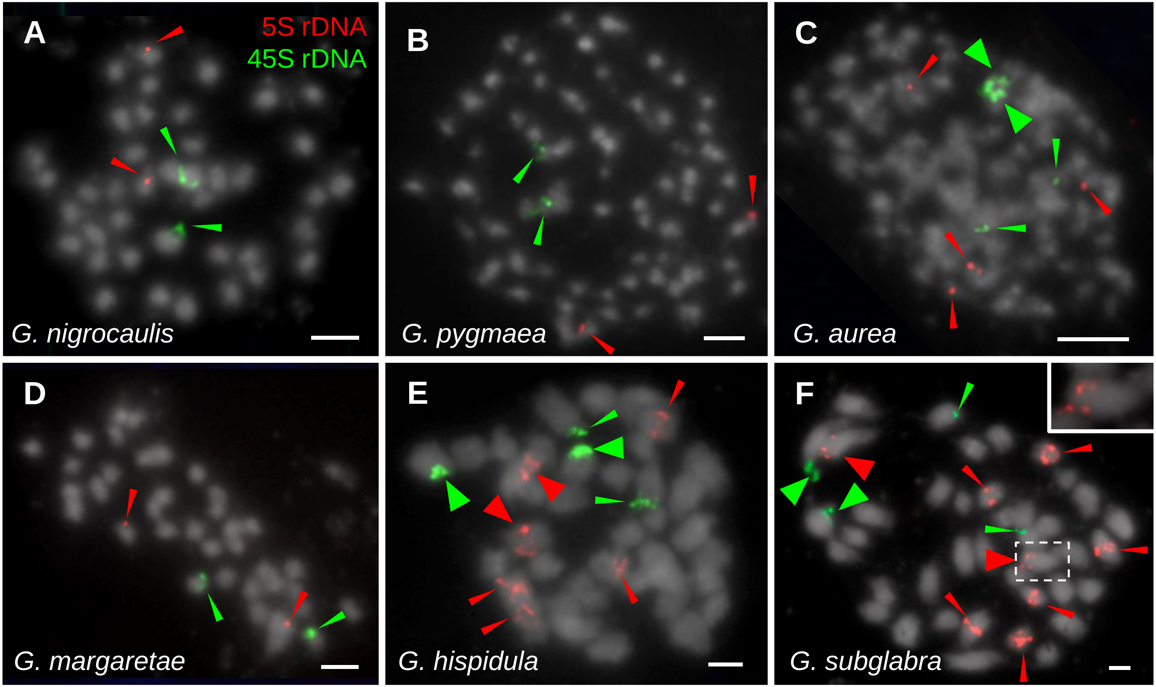

Supplementary figure 3: Different loci number and chromosomal distribution of 5S (in red) and 45S (in green) rDNA in *Genlisea* species. Bigger arrow heads denote chromosomes bearing two 5S rDNA loci (in red) or stronger 45S rDNA FISH signals (in green) in *G. aurea*, *G. hispidula* and *G. subglabra*. Bars = 3 µm.

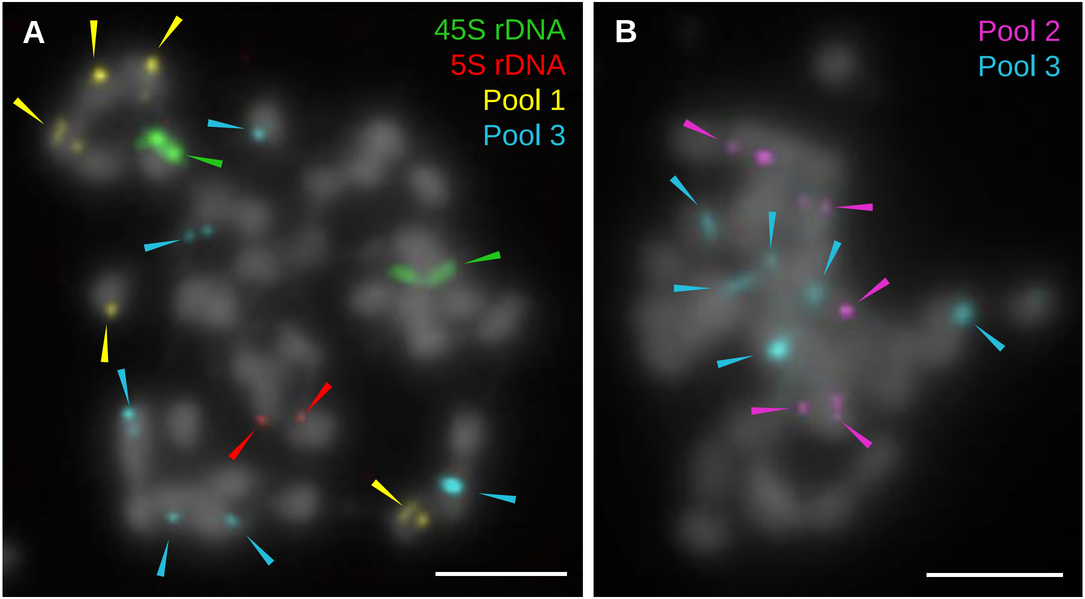

Supplementary figure 4: FISH confirming the separate localization of all three probes of pool 3 (cyan) in comparison to the rDNA (red and green) and pool 1 (yellow) (A) and pool 2 (magenta) (B). Each single-copy probe generated two FISH signals in a metaphase plate of *G. nigrocaulis*. However, one of them may be missing, especially in multicolor FISH combining different probes. Even the presence of only five separate signals (Figure 3I) for one pool is adequate for interpretation of separate chromosomal localization of three different single-copy probes as shown for pool 1 (**A**) and pool 2 (**B**). Bars = 3 µm.

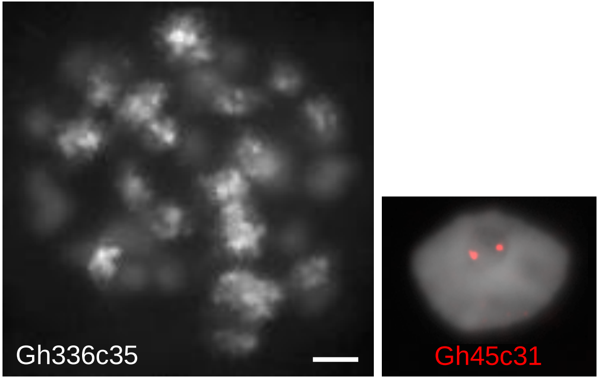

Supplementary figure 5: Cross-FISH using the Gh336c35 tandem repeat probe identified from *G. hispidula* yielded similar hybridization pattern on metaphase plate of *G. subglabra*. The Gh45c31 repeat showed on all analyzed flow-sorted nuclei one locus instead of two loci as revealed in *G. hispidula* (Figure 4G). Bar = 3 µm.

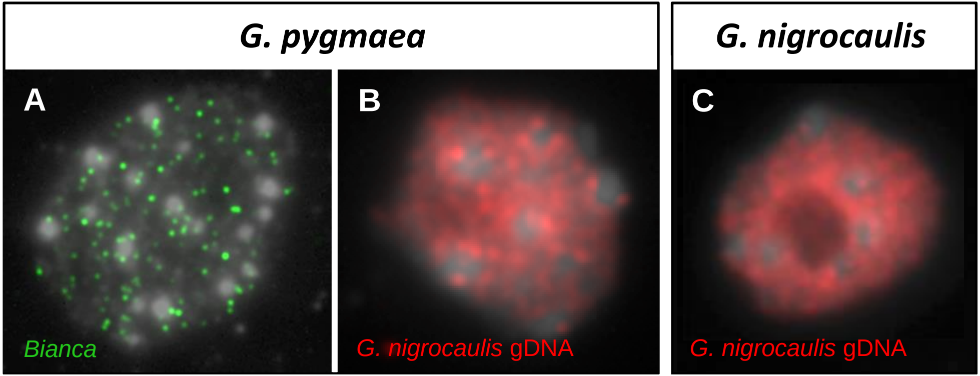

Supplementary figure 6: Cross-FISH revealed similar hybridization patterns in interphase nuclei of *G. nigrocaulis* and *G. pygmaea*. (**A**) The highly abundant *Ty1/copia* element *Bianca* of *G. nigrocaulis* yielded many signals throughout *G. pygmaea* interphase nuclei. FISH using *G. nigrocaulis* genomic DNA (gDNA) as probe in the presence of a 10-fold excess of unlabeled Gn7c161 repeat as blocking DNA resulted in homogenously distributed signals except at heterochromatin in interphase nuclei of *G. pygmaea* (**B**) and *G. nigrocaulis* (**C**).
